# The magnitude of suicidal ideation, attempts and associated factors of HIV positive youth attending ART follow ups at St. Paul’s hospital Millennium Medical College and St. Peter’s specialized hospital, Addis Ababa, Ethiopia, 2018

**DOI:** 10.1371/journal.pone.0224371

**Published:** 2019-11-05

**Authors:** Mesele Wonde, Haregewoin Mulat, Addis Birhanu, Aynalem Biru, Tilahun Kassew, Shegaye Shumet

**Affiliations:** 1 Department of Psychiatry, University of Gondar, Gondar, Ethiopia; 2 Amanuel Mental Specialized Hospital, Addis Ababa, Ethiopia; University of Toronto, CANADA

## Abstract

**Background:**

Suicide which is considered a psychiatric emergency, is a serious cause of mortality worldwide. Youth living with HIV/AIDS (YLWHA) have higher rates of suicidal behavior than the general public. This study aimed to assess the magnitude and associated factors of suicide ideation and attempt among the Human Immune deficiency Virus (HIV) positive youth attending anti-retroviral therapy (ART) follow up at St. Paul`s hospital Millennium Medical College and St. Peter`s specialized hospital, Addis Ababa, Ethiopia.

**Methods:**

In this cross-sectional study, 413 HIV positive youth were recruited for interviews, using the systematic random sampling technique. The Composite International Diagnostic Interview (CIDI) was used to assess suicide. PHQ-9, the Oslo social support and HIV perceived stigma scale instruments were used to assess the factors. We computed bivariate and multivariable binary logistic regressions to assess factors associated with suicidal ideation and attempt. Statistical significance was declared at P-value <0.05.

**Result:**

The magnitude of suicidal ideation and attempts were found to be 27.1% and 16.9%, respectively. In the multivariate analysis, female sex (adjusted odd ratio(AOR) = 3.1, 95% CI, 1.6–6.0), family death (AOR = 2.1, 95%CI 1.15–3.85), WHO clinical stage III of HIV (AOR = 3.1 95% CI 1.3–7.35), WHO clinical stage IV of HIV (AOR = 4.76, 95%CI, 1.3–7.35), co-morbid depression (AOR = 7.14, 95%CI, 3.9–12.9), and perceived HIV stigma (AOR = 4.2, 95%CI, 2.27–8.2) were significantly associated with suicidal ideation, whereas female sex (AOR = 4.12, 95%CI, 1.82–9.78), opportunistic infections (AOR = 3.1, 95%CI, 1.6–6.04), WHO clinical stage III of HIV (AOR = 3.1 95%CI 1.24–7.81), co-morbid depression (AOR = 5.6 95% CI, 2.8–11.1), and poor social support (AOR = 3.4, 95%CI, 1.2–9.4) were statistically significant with suicidal attempt. The result suggests that the magnitude of suicidal ideation and attempts among HIV positive youth were high. We recommend that clinicians consider youth with comorbid depression, perceived HIV stigma and poor social support.

## Introduction

HIV/ AIDS is one of the most serious health and development challenges in the world. About 36.7 million people were living with HIV at the end of 2016[1], of those 11.8 million were young people aged 15 to 24 years [2]. Approximately two-thirds of the victims were in sub-Saharan Africa, with 43% new cases in eastern and southern Africa. As HIV primarily affects the most productive age groups, one third of the new cases are from 15–24 years of age [3]. Youth and young adults account for a large percentage of the HIV/AIDS cases in Ethiopia. AIDS death has risen among adolescents and young men and women since 2001[4]. HIV epidemic has slowed economic growth and over all development in Africa by depleting the human resource capital [5] and erasing earlier gains life in expectancy [6, 7].

Mental health and HIV/AIDS are closely interlinked with each other; mental health problems increase the risk for HIV/AIDS and interferes with its treatment for lack of insight about the illness and decision-making problems. Conversely some mental disorders occur because of HIV infection which may result from HIV- related stigma, opportunistic infections and medication side effects [8, 9]. Because of the presence of advanced therapy that has transformed HIV/AIDS into a chronic illness, it is associated with high risk for suicidal ideation, attempted and completed compared to the general population [10]. Mental illness associated outcomes, such as suicide are a neglected global health priorities, particularly for the young public and a leading cause of health-related disability, affecting 10–20% of the youth with HIV worldwide [11].

Suicidal behavior is a complicated process that ranges in severity from thinking about killing oneself to doing it. Suicidal ideation is an important phase in the suicidal process, preceding attempted suicide; it is the major risk factor for completing suicide and is also a potentially fatal event. Suicide attempts are up to 20 times more frequent than complete suicides [12]. Suicidal behavior is a major health concern worldwide, and the problem varies across developed and developing countries. At least a million people are estimated to die annually by suicide worldwide [13, 14].

In the general public, the annual global suicide rate is 11.4 per 100 000 population or one death every 40 seconds. It is estimated to contribute over 2.4% of the global burden of disease by the year 2020, and the rate of death because of suicide will increase to one every 20 seconds [15]. The national cost of suicide, including suicide attempts, in the United States in 2013 was $58.4 billion [16, 17]. Suicide is now the 10th leading cause of death worldwide and the 3^rd^ leading cause of death between the ages 15–24 years [12].

People with HIV/AIDS have 7 to 36 times more risk for suicidal ideation and attempt compared to the general public [10, 18]. The pattern of suicide attempt and suicidal ideation may differ throughout the progression of HIV infection. The initial 6 months after the diagnosis of HIV and the onset of a physical complication of AIDS are a high risk period for suicidality [19]. Different studies showed that the global magnitude of suicidal ideation and attempts among HIV positive youth varied across the world. For example, the magnitude of suicidal ideation has been 10% in southeastern United States [20], 21.6% Chicago city, in USA [21], 14% in Canada [22], 15.5% in Thailand [23], 9.7% in Jamaica [24], 24% in Johannesburg [25], 16% in Nigeria [26], and 11% in Rwanda [27]. The magnitude of suicidal attempt was 9% in Japan[28], 3.5% in Canada[22], 8.2% in Thailand [23], 5% in Johannesburg, South Africa [25],1.3% in Nigeria [26], 20% in Rwanda [29], and Uganda [30].

Moderating and risk factors for suicide ideation and attempts among youth living with HIV/AIDS have been sex, depression, clinical stage of HIV, death in a family and hospital admission. HIV related stigma and poor social support have also been the other risk factors for suicide [20, 31–34].

Youth and young adults account for a large percentage of all HIV/AIDS cases in Ethiopia. Living with chronic diseases, like HIV/AIDS may increase the risk for suicide. Although suicide is common among HIV positive youth worldwide, there is little report about suicide among HIV positive youth in Ethiopia. So, the aim of this study was determining the magnitude of suicidal ideation and attempts and associated factors among HIV positive youth would contribute to early intervention and further decrease of the burden of suicide and to improve patients quality of life.

## Methods and materials

### Study settings and populations

An institution-based cross-sectional study was conducted among HIV positive youth attending ART follow ups at St. Paul’s hospital Millennium Medical College and St. Peter`s specialized hospital, Addis Ababa, Ethiopia, in May and June 2018. Addis Ababa, the capital of Ethiopia, had a total of 608 health facilities (hospitals, health centers, and private clinics) serving more than three million inhabitants. St. Paul’s hospital Millennium Medical College and St. Peter`s specialized hospital provide a variety of services, including Antiretroviral Therapy to adults and the youth.

### Participants

The participants of this study were HIV/AIDS patients receiving follow up care at St. Paul’s Millennium Medical College and St. Peter`s specialized hospitals, Addis Ababa, Ethiopia. The single population proportion formula, $n=\frac{{\left(Z\frac{\alpha }{2}\right)}^{2}p(1-p)}{d^{2}}$ with 5% margin of error, 95% confidence level and 50% proportion used to calculate sample size yielded 423 (including 10% non-response rate). The average number of patients was calculated with their monthly visit in mind. Participants were selected for interviews using the systematic random sampling technique.

### Inclusion and exclusion criteria

HIV positive youth attending ART follow ups in both hospitals during the study were included, whereas participants seriously ill and unable to communicate were excluded.

### Measurement

Suicidal ideation and attempts were assessed using the suicidality module of the World Mental Health (WMH) survey initiative version 3.0 of the World Health Organization (WHO) Composite International Diagnostic Interview (CIDI) which was validated in Ethiopia. To assess suicidal ideation, participants were asked: “Have you ever seriously thought about committing suicide?” If “yes”, the patient had suicide ideation. For suicidal attempts, participants were asked: “Have you ever attempted suicide?” if “yes”, the patient had made a suicide attempts [35]. Depression was measured by PHQ-9. A cut off point of ten and above was used for depression [36]. Social support was measured by the Oslo social support scale. The scale ranged from 3–14,and the scores 3–8,9–11,and 12–14 stood for “poor”, “moderate” and “strong” social support, respectively [37]. Stigma was measured by a 12 item HIV perceived stigma scale. The scale consisted of a four-point likert scale questions concerning disclosure status, negative self-image, and public attitudes to which the responses varied from “strongly disagree” to “strongly agree”. Patients who scored greater than or equal to the mean (≥19.7) were considered as having HIV perceived stigma [38]. To assess current substance use, respondents were asked: “Have you ever used any substance (none medical use) for the last three months?”, and forever use of substance, respondents were asked,” Have you ever used substance (none medical use) in your lifetime?”

Data on stages of HIV, CD4 count, drug regimen and history of opportunistic infections were gathered from patient records.

Items on socio-demographic factors (age, sex, ethnicity, religion, marital status, educational status and occupational status) were adopted from a variety of literature[20, 34].

### Data collection

Data were collected by face to face interviews using a semi-structured questionnaire by four trained psychiatry nurses by the Amharic version of the tool for a month. The questionnaire was designed in English and translated to Amharic and back to English to maintain consistency. Data collectors were trained on an introduction to suicide and HIV, research methods, interviewing skills, sampling, recruitment and the ethical aspects of the research.

### Data processing and analysis

All data were checked for completeness and consistency and entered in to Epi-Data version 3.1 and then exported to SPSS for windows version 20 for analysis. Descriptive and bivariate logistic regression analyses were computed to see the frequency distribution and to test the association between independent and dependent variables, respectively. Factors associated with suicidal ideation and attempts were selected during the bivariate analysis with a p-value ≤0.2 for further multivariate analysis in which variables with less than 0.05 P-value at a 95% confidence interval were considered as statistically significant.

### Ethical considerations

Approval was obtained from the Ethical Review Board and ethical clearance from the joint Ethical Review Committees of the University of Gondar and Amanuel mental specialized hospital. A formal letter of permission was obtained from St. Paul’s hospital Millennium Medical College and St. Peter`s specialized hospital. We received written informed consent from the study participants and assent from officially authorized foster parents after explaining the purpose of the study. Confidentiality was maintained by omitting personal identifiers.

## Results

A total of 413 participants took part with a response rate of 97.6%. The mean age of the respondents was 20.9 (±SD = 2.9) years; more than three-fifths (63.2%), of were female; 235 (56.9%) were Orthodox Christian; 331 (80.1%) were single, and 174(42.1%) Amhara by ethnicity. More than half (54%) had primary school education, and 137(33.2%) had history of family death. Of the respondents, 334(80.9%) lived with their family members, and about 360(87.2%) disclosed their HIV status (**Table 1**).

**Table 1:** Sociodemographic characteristics of HIV positive youth follow up at SPSH and SPHMMC Addis Ababa, Ethiopia, 2018 (n = 413).

Variables	Category	Frequency(n)	Percentage (%)
Age	15–19	131	31.7
	20–24	282	68.3
Sex	Male	152	36.8
	Female	261	63.2
Religion	Orthodox	235	56.9
	Muslim	109	26.4
	Protestant	69	16.7
Ethnicity	Amhara	174	42.1
	Oromo	156	37.8
	Tigre	18	4.4
	Gurage	65	15.7
Marital status	Married	67	16.2
	Single	331	80.1
	Divorced	5	1.2
	Widowed/er	10	2.4
Educational status	Uneducated	31	7.5
	primary	223	54
	secondary	58	14
	preparatory	36	8.7
	college and above	65	15.7
Family death	No	276	66.8
	Yes	137	33.2
Living arrangement	With family	334	80.87
	Alone	79	19.13
Disclosure status	Yes	360	87.2
	No	53	12.8

Of the respondents, 187(45.3%) were on WHO clinical stage I of HIV/AIDS. The majority, 376(91%) had CD4 count ≥200 cell/mm3, and about 154(37.3%) had history of opportunistic infections. A small number, 28(6.8%) and 16(3.9%), of the participants had family history of mental illness and suicide, respectively. Nearly one-third (31.7%) of the respondents had comorbid depression symptoms (**Table 2**).

**Table 2:** Clinical factors of HIV positive youth follow up at SPSH and SPHMMC Addis Ababa, Ethiopia, 2018 (n = 413).

Variables	Category	Frequency(n)	Percentage (%)
WHO clinical stage of HIV	Stage I	187	45.3
	Stage II	137	33.2
	Stage III	48	11.6
	Stage IV	41	9.9
CD4 count	<200 cells/mm3	37	9
	≥200 cells /mm3	376	91
Serum status knowing duration	<6month	14	3.4
	> = 6 month	399	96.6
Opportunistic infections (OI)	No	259	62.7
	Yes	154	37.3
Family history of mental illness	No	385	93.2
	Yes	28	6.8
Family history of suicide	No	397	96.1
	Yes	16	3.9
History of hospital admission	No	329	79.7
	Yes	84	20.3
Type of medication	1e (TDF-3TC-EFV)	253	61.3
	1d (AZT-3TC-EFV)	105	25.4
	1c (AZT-3TC-NVP)	26	6.3
	1f (TDF+3TC+NVP)	17	4.1
	*Others	12	3
Depression	No	282	68.3
	Yes	131	31.7

*others = 2a, 2e, 1h

**Fig 1:**
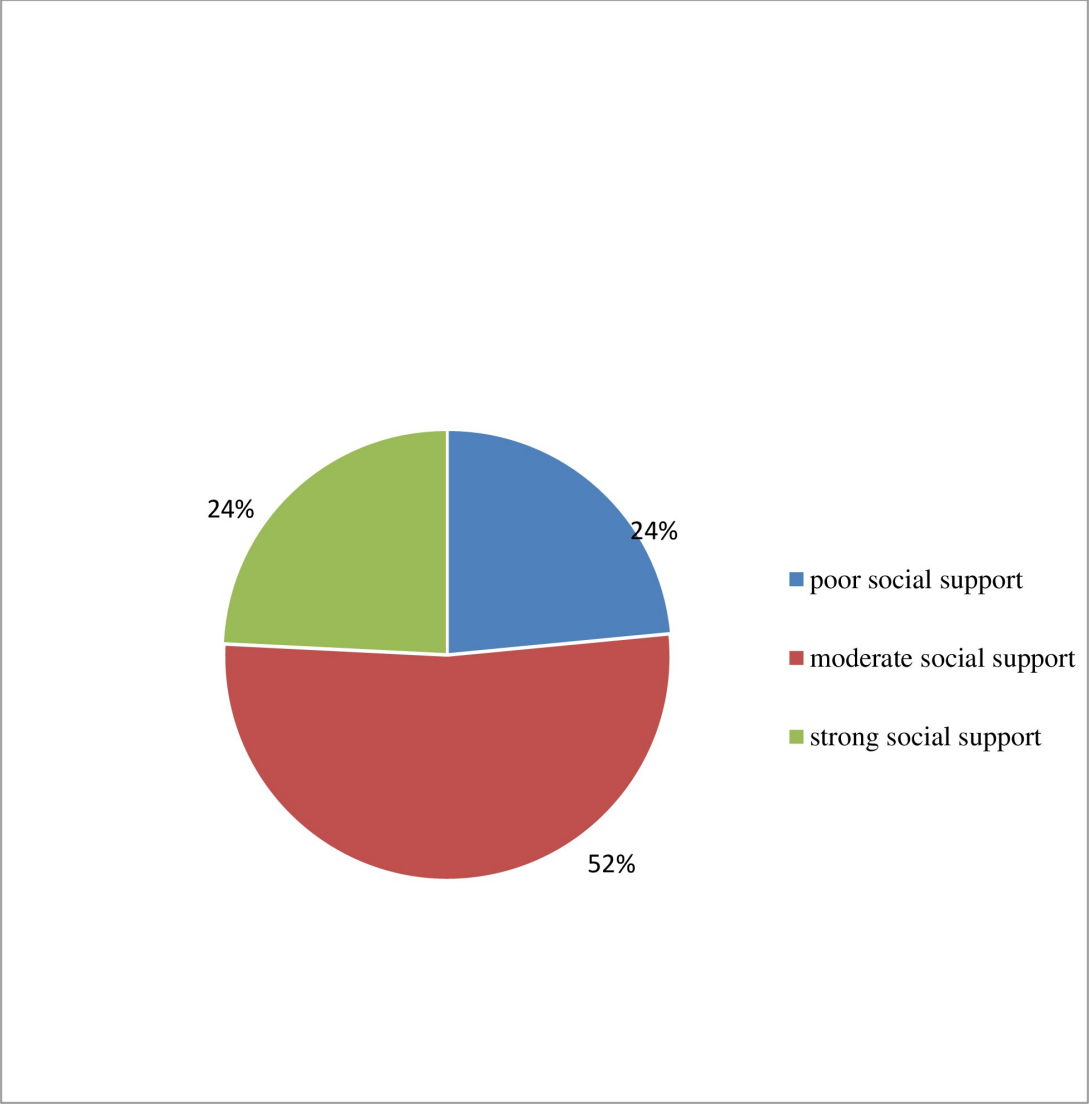
Social support of HIV positive youth at SPSH and at SPHMMC Addis Ababa, Ethiopia, 2018 (n = 413).

Regarding psychosocial factors, more than half (52.3%) and 47.7% of the participants had perceived HIV stigma and no such stigma, respectively. About ninety-seven participants had poor social support (**Fig 1**).

**Fig 2:**
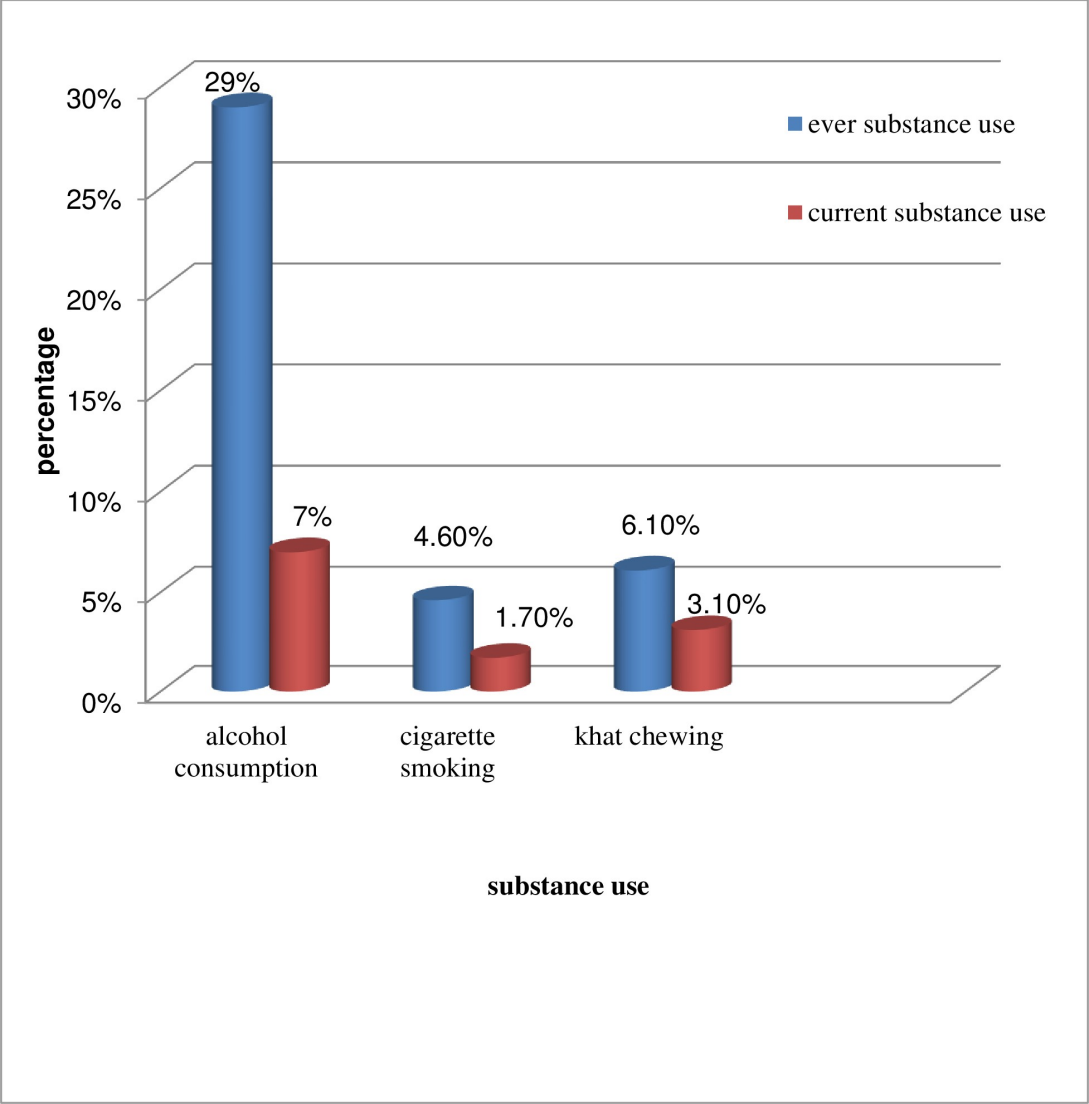
Substance-related characteristics of HIV positive youth follow up at SPSH and SPHMMC Addis Ababa, Ethiopia, 2018 (n = 413).

Of the participants, 121 (29.3%) consumed alcohol once in their lifetime, and 25(6.5%) were doing that at the moment; 13(3.1%) used khat (leaves) /Catha edulis/during the study (**Fig 2**).

### Magnitude of suicidal ideation and attempt

The magnitude of suicidal ideation and suicidal attempt among participants were 27.1%, with a 95% CI (22.5%, 31.5%) and 16.9% with a 95% CI (13.3%, 20.3%), respectively. More than three fourths, 54 (77.1%) attempted suicide once in their lifetime, and 44(62.9%) used drug over-dose for the attempts (**Table 3**).

**Table 3:** Suicidal ideation and attempt among respondents at SPSH and SPHMMC Addis Ababa, Ethiopia, 2018 (n = 413).

Variables	Category	Frequency (n)	Percent (%)
Ever suicidal ideation	No	301	72.9
	Yes	112	27.1
Suicidal ideation in one month	No	391	94.3
	Yes	22	5.7
Ever plan of suicide	No	356	86.2
	Yes	57	13.8
Ever suicide attempt	No	343	83.1
	Yes	70	16.9
Suicide attempt in one month	No	399	96.6
	Yes	14	3.4
Frequency of suicide attempt	Once	54	77.1
	Two times	9	12.9
	More than two	7	10
Reason for suicidal attempt	Physical illness	52	72.9
	Family conflict	16	22.9
	Death of family/poverty	3	4.3
Method of attempt	Hanging	18	25.7
	Drug over dose	44	62.9
	Poisoning	8	11.4

### Factors associated with suicidal ideation among HIV positive youth

To determine the association of independent variables with suicidal ideation, bivariate and multivariate logistic regression analyses were carried out. In the bivariate analysis, factors including female sex, history of family death, WHO clinical stage of HIV, opportunistic infections, living arrangements, disclosing HIV status, depression, social support, perceived stigma to HIV, and family history of mental illness were significantly associated with suicidal ideation. The result of the multivariate analysis showed that female sex, WHO clinical stage of HIV, history of family death, comorbid depression and perceived HIV stigma were statistically significant with suicidal ideation at a p-value <0.05 (**Table 4**).

**Table 4:** Factors associated with suicidal ideation of participants at St. SPSH and SPHMMC Addis Ababa, Ethiopia, 2018 (n = 413).

Variables	Category	Suicidal ideation		COR, (95%CI)	AOR, (95%CI)
		No	yes		
Sex	Male	130	22	1	1
	Female	171	90	3.11(1.9–5.2)	3.1(1.6–6.0)***
History of a family death	No	215	61	1	1
	Yes	86	51	2.1(1.3–3.3)	2.1(1.2–3.9)*
WHO clinical stage of HIV	Stage I	152	35	1	1
	Stage II	106	31	1.3(.7–2.2)	1.5(.7–2.9)
	Stage III	29	19	2.9(1.4–5.7)	3.1(1.3–7.4)*
	Stage IV	14	27	8.4(4.0–17.6)	4.8(1.8–1.8)**
Opportunistic infection	No	199	60	1	1
	Yes	102	52	1.7(1.1–2.6)	1.18(.7–2.2)
Living arrangement	With family	268	72	1	1
	Alone	33	40	4.5(2.7–7.7)	1.2(.5–3.1)
Disclose of HIV status	Yes	282	78	1	1
	No	19	34	6.5(3.5–11.9)	2.7(.9–7.7)
Depression	No	248	34	1	1
	Yes	53	78	10.7(6.5–17.7)	7.1(3.9–12.9)***
Social support	Poor	56	41	4.5(2.3–9.1)	1.2(.5–2.9)
	Moderate	159	57	2.2(1.2–4.2)	1.3(.6–2.8)
	Strong	86	14	1	1
HIV perceived stigma	No	174	23	1	1
	Yes	127	89	5.3(3.2–8.9)	4.3(2.3–8.2)***
History of family mental illness	No	285	100	1	1
	Yes	16	12	2.1(.9–4.7)	0.7(.2–2.1)

*P- value < 0.05

** *P*-value < 0.01

***P-value < 0.001, VIF = 1.06–2.1

Goodness of fit test corresponding P-value = 0.68

### Factors associated with suicide attempts among HIV positive youth

In the bivariate logistic regression factors including female sex, history of opportunistic infection, WHO clinical stage III of HIV, living arrangements, disclosure status of HIV,depression, social support, perceived stigma to HI, family history of mental illness, and history of family death were significantly associated with suicidal attempts.

The result of the multivariate analysis showed that female sex, history of opportunistic infections, WHO clinical stage III of HIV, comorbid depression and poor social support were statistically significant to suicidal attempts (**Table 5**).

**Table 5:** Factors associated with a suicide attempt of participants at SPSH and SPHMMC Addis Ababa, Ethiopia, 2018 (n = 413).

Variables	Category	Suicidal attempt		COR (95%CI)	AOR (95%CI)
		No	yes		
Sex	Male	142	10	1	1
	Female	201	60	4.2 (2.1–8.6)	4.1(1.8–9.8)**
WHO clinical stage HIV	Stage I	164	23	1	1
	Stage II	124	13	.8(.4–1.5)	.6(.3–1.4)
	Stage III	33	15	3.2(1.5–6.9)	3.1(1.2–7.8)*
	Stage IV	22	19	6.2(2.9–13.1)	2.5(.9–6.4)
Opportunistic infection	No	233	26	1	1
	Yes	110	44	3.6 (2.1–6.1)	3.1(1.6–6.0)**
Living arrangement	With family	294	46	1	1
	Alone	49	24	3.1 (1.8–5.6)	.8(.3–2.3)
Disclose of HIV status	Yes	311	49	1	1
	No	32	21	4.2(2.2–7.8)	1.7(.5–5.5)
Depression	No	263	19	1	1
	Yes	80	51	8.8(4.9–15.8)	5.6(2.8–11.1)***
Social support	Poor	61	36	5.9(2.7–13.3)	3.4(1.2–9.4)*
	Moderate	191	25	1.3(.6–2.9)	1.0(.4–2.6)
	Strong	91 9	9	1	1
HIV perceived stigma	No	177	20	1	1
	Yes	166	50	2.7 (1.5–4.7)	1.6(.8–3.4)
History of family mental illness	No	323	20	1	1
	Yes	62	8	2.1(.9–4.9)	.9(.3–2.9)
History of family death	No	236	107	1	1
	Yes	40	30	1.7(.9–2.8)	1.1(.6–2.2)

*P-value < 0.05

** *P*-value < 0.01

***P-value < 0.001, VIF 1.06–2.10

Goodness of fit test corresponding P-value = 0.37

## Discussion

Youth living with HIV/AIDS had high rates of suicidal behavior. The magnitude of suicidal ideation among HIV positive youth was 27.1% with a 95% CI (22.5%-31.5%). This finding was consistent with reports of other studies. For example,31% in Uganda [39], 24% in South Africa [25] and 26% in USA [40]. Conversely, our 27.1% was higher than the results of various studies, such as 16% in Nigeria [26], 11% in Rwanda [27], 15.5% in Thailand [23], 9.7% in Jamaica, [24], 11.5% in Mexico [41], 14.0% in Canada [22] and 21.6%, and 10.0% in USA [21, 42].The possible reason for the differences might be small sample size and population variations; for example, in Nigeria, children were included in the study [26]. It might also be variations in the health status of the study population, for instance HIV negative youth participated in Jamaica, Mexico and Canada, whereas this study focused on HIV-positive youth. Furthermore, study design (retrospective, prospective cohort) conducted in USA and a case-control in Rwanda and Thailand might have been the other possible reasons.

On the other hand, this finding was lower than those of other countries, like 31.6 and 64% in China [33, 43] where a cohort study was carried out. It might also be due to socio-cultural differences, for example, the degree of openness with which people reported their experiences might not be similar across cultures.

The magnitude of suicidal attempt in this study was 16.9% with a 95% CI (13.3%-20.3%), consistent with those of other studies, for example, 19.5%, 20% in Kampala and Uganda [30, 39, 44] and 20% in Rwanda[29]. Suicidal attempts in this study were higher than the 12% noted in Rwanda [27], 1.3% in Nigeria [26], 5% in South Africa [25], 8.2% in Thailand [23], 9% in Japan [28] and 13% in US [42]. The difference might be variations in the study populations. For example, in the Nigerian study children were included. Suicide attempts are rare before 12 years of age due to cognitive immaturity as a protective factor [12]. Another possible reason might be differences in the health status of study populations involved. For example, HIV negative youth participated in Japan, while our work focused on HIV-positive youth. Prospective cohort study designs used in the US and the case-control study in Rwanda and Thailand might have also been other possible reasons for the variations. But 16.9% was lower than 22.6% in China [33]. This difference might be the result of socio-cultural variations relating to participants’ readiness to disclose their experience of suicidal attempts openly.

Female sex, history of family death, WHO clinical stage III and IV of HIV, comorbid depression, and perceived stigma about HIV were significantly associated with suicidal ideation. The greater likelihood of suicidal ideation among women than men in our work was similar to the reports of other studies in Jamaica [24], Mexico [41], South Africa [45] and the US [42], possibly because of women’s greater vulnerability to psychological distress due to difference hypotheses involving hormonal differences, the effects of childbirth, psychosocial stressors and behavioral models of learned helplessness[12]. Suffering from different psychological distress, might lead to suicidal ideation because they may consider suicide a better choice for ending their emotional pain, and results might lead to suicide ideation.

Participants with history of family death were more likely to develop suicide ideation than those who had no history family death. This result is supported by other studies, in South Africa[46] and America [47].

WHO clinical stages of III and IV of HIV were significantly associated with suicidal ideation. This might be because the classification of clinical stages are based on the presence and absence of opportunistic infections. HIV-positive patients on advanced clinical stages might have poor quality of life which may lead them to think of death. This views is supported by other studies [48, 49]. Comorbid depression was found to be significantly associated with suicidal ideation. This is consistent with the findings of studies in Japan [28] and China [33]. This is probably because depressed individuals might have low serotonin neurotransmitter. This neurotransmitter disturbance in the brain might contribute to hopelessness, guilt, and worthlessness which might again expose to suicidal ideation [50].

In addition, perceived stigma for HIV was significantly associated with suicidal ideation. This result is in line with the findings of other studies in South Africa [46], China [33], and America [47]. In this study, almost half (52.3%) of the youth experienced HIV related perceived stigma which might be related to stigma attributed to psychological distress, low self-esteem, anxiety and depression [51]. The negative effects of stigma may aggravate the psychological pressure of youth living with HIV/AIDS and might lead to suicidal ideation because they might consider suicide as a better choice for ending their emotional pain and discrimination resulting from the disease.

Female sex, opportunistic infections, WHO clinical stages of HIV, comorbid depression and poor social support were significantly associated with suicide attempts. Females were more likely to develop suicidal attempts than males as reported in South Africa, Canada and the US [42, 45, 52]. Women have greater vulnerability to psychological distress because of various hypothetical hormonal differences, the effects of childbirth, psychosocial stressors, and behavioral models of learned helplessness[12], which might push them to consider suicide a better choice for ending their emotional pain and make suicide attempts.

Patients with opportunistic infections were 3.1 times more likely to attempt suicide compared to patients with no such infections. This is possibly because physically weak and emaciated patients might be too hopeless to make suicide attempts. The current result is consistent with those of other studies [34].

WHO clinical stage of HIV was significantly associated with suicidal attempts. HIV positive patients with advanced immune suppression may suffer from a variety of opportunistic infections, and the agony might lead them to suicidal attempts. This finding was supported by the results of other works[48]. In this study patients with comorbid depressive symptoms were high likely to make suicidal attempts. It might be the direct effect of depression on patients to feel hopeless, isolated and worthless [50].This is consistent with the result of a previous study in Japan [28].

Social support plays an important role in the psychological adjustment of people living with HIV/AIDS[53]. Patients with poor social support may face difficulty adjusting to psychological problem by themselves and feel lonely to the extent of increasing their suicidal risk [54]. In this study patients with poor social support were at high risk for suicidal attempts.

### Limitation of the study

Our cross-sectional design has prevented us from reporting the casual relationships of the associations we found. In addition, social desirability and recall bias might have also been the other limitations. Because the data collection method was face-to-face interviews, individuals might have given socially acceptable answers during the interviews, especially to substance-related questions.

## Conclusion

The magnitude of suicidal ideation and attempts among youth living with HIV/AIDS were found to be high. Both suicidal ideation and attempts were statistically significant with the female sex, WHO clinical stage of HIV and co-morbid depression. History of family death and perceived HIV stigma were significantly associated with suicidal ideation. The presence of opportunistic infections and poor social support were significantly associated with suicidal attempts. Therefore, we recommend early suicide focused regular screening and linkage with mental health service providers. It is necessary to give emphasis to youth with comorbid depressive symptoms, perceived HIV stigma and the treatment of opportunistic infections (**S1 Table and S2 Table**).

## Supporting information

S1 Table Factors associated with suicidal ideation of participants at St. SPSH and SPHMMC Addis Ababa, Ethiopia, 2018 (n = 413). (PDF)

S2 Table Factors associated with a suicide attempt of participants at SPSH and SPHMMC Addis Ababa, Ethiopia, 2018 (n = 413). (PDF)

## Abbreviations

AMSH: Amanuel Mental Specialized Hospital
AIDS: Acquired Immune Deficiency Syndrome
CD4: Cluster of differentiation 4
HIV: Human Immunodeficiency Virus
MINI: Mini International Neuropsychiatric Interview
OSS: Oslo Social Support
PHQ-9: Patient Health Questioner 9
SPHMMC: Sent Paul Hospital Millennium Medical College
SPSH: Sent Peter Specialized Hospital
WHO: World Health Organization
