# Supplementary material for: The magnitude of suicidal ideation, attempts and associated factors of HIV positive youth attending ART follow ups at St. Paul’s hospital Millennium Medical College and St. Peter’s specialized hospital, Addis Ababa, Ethiopia, 2018
Source: PLoS One. 2019 Nov 5;14(11):e0224371. doi: 10.1371/journal.pone.0224371 (PMC6830816; doi:10.1371/journal.pone.0224371)
Supplement: S1 Table — (PDF) [file pone.0224371.s001.pdf]

| Variables | Category | Suicidal ideation | | COR, (95%CI) | AOR, (95%CI) |
|---|---|---|---|---|---|
| | | No | yes | | |
| Sex | Male | 130 | 22 | 1 | 1 |
| | Female | 171 | 90 | 3.11(1.9-5.2) | 3.1(1.6-6.0)*** |
| History of a family death | No | 215 | 61 | 1 | 1 |
| | Yes | 86 | 51 | 2.1(1.3-3.3) | 2.1(1.2-3.9)* |
| WHO clinical stage of HIV | Stage I | 152 | 35 | 1 | 1 |
| | Stage II | 106 | 31 | 1.3(.7-2.2) | 1.5(.7-2.9) |
| | Stage III | 29 | 19 | 2.9(1.4-5.7) | 3.1(1.3-7.4)* |
| | Stage IV | 14 | 27 | 8.4(4.0-17.6) | 4.8(1.8-1.8)** |
| Opportunistic infection | No | 199 | 60 | 1 | 1 |
| | Yes | 102 | 52 | 1.7(1.1-2.6) | 1.18(.7-2.2) |
| Living arrangement | With family | 268 | 72 | 1 | 1 |
| | Alone | 33 | 40 | 4.5(2.7-7.7) | 1.2(.5-3.1) |
| Disclose of HIV status | Yes | 282 | 78 | 1 | 1 |
| | No | 19 | 34 | 6.5(3.5-11.9) | 2.7(.9-7.7) |
| Depression | No | 248 | 34 | 1 | 1 |
| | Yes | 53 | 78 | 10.7(6.5-17.7) | 7.1(3.9-12.9)*** |
| Social support | Poor | 56 | 41 | 4.5(2.3-9.1) | 1.2(.5-2.9) |
| | Moderate | 159 | 57 | 2.2(1.2-4.2) | 1.3(.6-2.8) |
| | Strong | 86 | 14 | 1 | 1 |
| HIV perceived stigma | No | 174 | 23 | 1 | 1 |
| | Yes | 127 | 89 | 5.3(3.2-8.9) | 4.3(2.3-8.2)*** |
| History of family mental illness | No | 285 | 100 | 1 | 1 |
| | Yes | 16 | 12 | 2.1(.9-4.7) | 0.7(.2-2.1) |

**P*- value < 0.05, ** *P*-value < 0.01, and ****P*-value < 0.001, VIF=1.06-2.1

Goodness of fit test corresponding P-value = 0.68
