# Supplementary material for: The magnitude of suicidal ideation, attempts and associated factors of HIV positive youth attending ART follow ups at St. Paul’s hospital Millennium Medical College and St. Peter’s specialized hospital, Addis Ababa, Ethiopia, 2018
Source: PLoS One. 2019 Nov 5;14(11):e0224371. doi: 10.1371/journal.pone.0224371 (PMC6830816; doi:10.1371/journal.pone.0224371)
Supplement: S2 Table — (PDF) [file pone.0224371.s002.pdf]

Variables	Category	Suicidal attempt		COR (95%CI)	AOR (95%CI)
		No	yes		
Sex	Male<br>Female	142<br>201	10<br>60	1<br>4.2 (2.1-8.6)	1<br>4.1(1.8-9.8)**
WHO clinical stage HIV	Stage I<br>Stage II<br>Stage III<br>Stage IV	164<br>124<br>33<br>22	23<br>13<br>15<br>19	1<br>.8(.4-1.5)<br>3.2(1.5-6.9)<br>6.2(2.9-13.1)	1<br>.6(.3-1.4)<br>3.1(1.2-7.8)*<br>2.5(.9-6.4)
Opportunistic infection	No<br>Yes	233<br>110	26<br>44	1<br>3.6 (2.1-6.1)	1<br>3.1(1.6-6.0)**
Living arrangement	With family<br>Alone	294<br>49	46<br>24	1<br>3.1 (1.8-5.6)	1<br>.8(.3-2.3)
Disclose of HIV status	Yes<br>No	311<br>32	49<br>21	1<br>4.2(2.2-7.8)	1<br>1.7(.5-5.5)
Depression	No<br>Yes	263<br>80	19<br>51	1<br>8.8(4.9-15.8)	1<br>5.6(2.8-11.1)***
Social support	Poor<br>Moderate<br>Strong	61<br>191<br>91 9	36<br>25<br>9	5.9(2.7-13.3)<br>1.3(.6-2.9)<br>1	3.4(1.2-9.4)*<br>1.0(.4-2.6)<br>1
HIV perceived stigma	No<br>Yes	177<br>166	20<br>50	1<br>2.7 (1.5-4.7)	1<br>1.6(.8-3.4)
History of family mental illness	No<br>Yes	323<br>62	20<br>8	1<br>2.1(.9-4.9)	1<br>.9(.3-2.9)
History of family death	No<br>Yes	236<br>40	107<br>30	1<br>1.7(.9-2.8)	1<br>1.1(.6-2.2)

**P*-value < 0.05, ** *P*-value < 0.01, and ****P*-value < 0.001, VIF 1.06-2.10

Goodness of fit test corresponding P-value = 0.37
